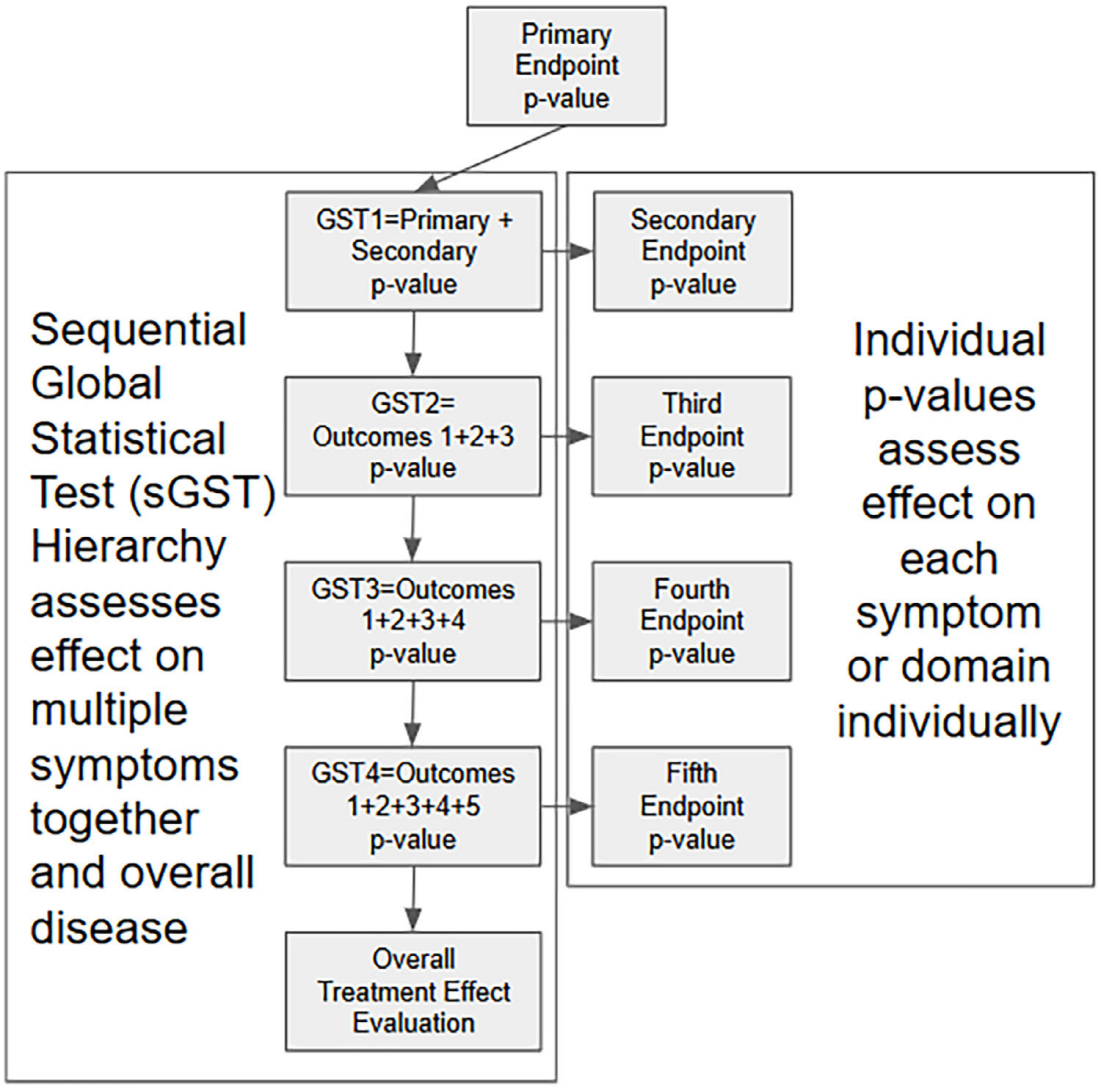# Sequential Global Statistical Tests: A Novel Method for Evaluating Primary, Secondary and Exploratory Variables in a Hierarchy with an Accumulated Evidence Approach

**DOI:** 10.1002/alz70859_105726

**Published:** 2025-12-25

**Authors:** Kent Hendrix, Craig Mallinckrodt, Suzanne B. Hendrix, Samuel P. Dickson

**Affiliations:** ^1^ Pentara Corporation, Salt Lake City, UT USA

## Abstract

**Background:**

Alzheimer’s disease (AD), is heterogeneous across patients and also highly variable from one day to the next. Cognition, function and global performance are measured with patient performance, a study partner questionnaire and an interview with a clinician. The high heterogeneity, subjectivity and different collection methods contribute to low correlations between clinical endpoints, in contrast to cardiovascular disease, for instance, with objective and consistent endpoints, resulting in higher correlations. Accurate interpretation of the evidence for a treatment effect relies on knowledge of these correlations, but they are rarely considered.

**Method:**

A novel statistical approach is proposed which parallels the informal process of reading through primary, secondary and exploratory results to assess the overall efficacy of a treatment. With each sequential endpoint, a reader assesses whether new information adds to or subtracts from the current evidence for a treatment effect, finally concluding whether a treatment works or not (See figure). Sequential Global Statistical Tests (sGSTs), can formally quantify the evidence for efficacy up to and including each outcome, accurately evaluating treatment efficacy. This sGST objectively accounts for redundancy and independence between endpoints using correlations, which are rarely considered in informal approaches. The utility of a cumulative sGST is demonstrated under simulated scenarios with high and low correlations.

**Result:**

Although correlations are rarely considered in an informal evaluation, they dramatically impact the interpretation of multivariate results. Expecting significance on secondary and exploratory endpoints to support a significant primary endpoint is only appropriate when the correlation between outcomes is very high. With low correlation, a secondary or exploratory endpoint showing a trend, or directional consistency can still support a significant primary endpoint. This leads to incorrectly negative conclusions in AD.

**Conclusion:**

The high heterogeneity of AD results in low correlations between endpoints, making it more prone to misinterpretation of overall treatment effects than diseases with higher correlations. Expecting significance on all outcomes is likely at least partly responsible for the high failure rate in AD. Using an sGST addresses this concern, by statistically ensuring no double counting of results with highly correlated outcomes, and no underestimation of the evidence provided by nearly independent outcomes.